# Psychological outcomes of low-dose CT lung cancer screening in a multisite demonstration screening pilot: the Lung Screen Uptake Trial (LSUT)

**DOI:** 10.1136/thoraxjnl-2020-215054

**Published:** 2020-10-21

**Authors:** Sonja Kummer, Jo Waller, Mamta Ruparel, Stephen W Duffy, Samuel M Janes, Samantha L Quaife

**Affiliations:** 1 Research Department of Behavioural Science and Health, University College London, London, UK; 2 School of Cancer and Pharmaceutical Sciences, King’s College London, London, UK; 3 Lungs for Living Research Centre, UCL Respiratory, Division of Medicine, University College London, London, UK; 4 Wolfson Institute of Preventive Medicine, Barts and the London School of Medicine and Dentistry, Queen Mary University of London, London, UK

**Keywords:** lung cancer, psychology

## Abstract

**Background:**

Previous studies of psychological burden in low-dose CT (LDCT) lung cancer screening trials may lack generalisability due to participation bias and control arms having elevated distress.

**Methods:**

Current and former smokers (n=787, aged 60–75) within a real-world screening demonstration pilot completed measures of lung cancer worry at three time points (T_0_: appointment, T_1_: next day, T_2_: 3 months) and anxiety and depression at two time points (T_0_ and T_2_). A ‘screening unaware’ community sample (n=383) with the same age and smoking characteristics completed these measures once (T_0_). Mean scores were compared by sample type and LDCT result.

**Results:**

Compared with the community sample (T_0_), mean scores were higher in the screening sample, and statistically significantly increased in adjusted analyses, for lung cancer worry at T_0_ and T_2_ (mean (M): 9.32; 95% CI 8.96 to 9.69 vs M: 11.34; 11.09 to 11.59 and M: 11.88; 11.49 to 12.27), for anxiety at T_0_ and T_2_ (M: 3.32; 2.94 to 3.70 vs M: 4.73; 4.42 to 5.04 and M: 5.78; 5.33 to 6.23) and depression at T_2_ (M: 3.85; 3.44 to 4.27 vs M: 4.15; 3.76 to 4.55). Scores were highest for those with indeterminate (eg, T_2_ anxiety M: 6.93; 5.65 to 8.21) and incidental findings (primary care follow-up M: 5.34; 4.67 to 6.02) and those ineligible for screening (M: 6.51; 5.25 to 7.77). Being female, younger, not in paid employment, not married/cohabiting with a partner and lower education predicted poorer psychological outcomes at T_0_, but not T_2_ after adjusting for baseline scores. Mean scores remained within ‘normal’ clinical ranges.

**Conclusion:**

Psychological distress was raised among high-risk individuals undergoing LDCT screening in a real-world setting, but overall differences were unlikely to be clinically meaningful. It will be critical to monitor the psychological impact of services longitudinally across diverse settings, including subgroups vulnerable to clinically elevated distress.

**Trial registration:**

The Lung Screen Uptake Trial was registered prospectively with the International Standard Registered Clinical/soCial sTudy (ISRCTN) (Number: ISRCTN21774741) on 23 September 2015 and the National Institutes of Health ClinicalTrials.gov database (NCT02558101) on 22 September 2015.

**Key messages**

**What is the key question?**

- Is there a clinically significant psychological impact of lung cancer screening when offered in a real-world setting and compared with ‘screening unaware’ individuals?

**What is the bottom line?**

- Psychological distress was raised among high-risk individuals undergoing low-dose CT screening in a real-world setting, particularly those with abnormal results or who were ineligible, but differences were unlikely to be clinically meaningful.

**Why read on?**

- This study reports the first real-word data on psychological outcomes from lung cancer screening using a sample representative of high-risk individuals; evidence crucial to informing decision-making about implementing lung cancer screening internationally.

## Introduction

Lung cancer leads cancer-related mortality worldwide, with 35 148 deaths recorded in the UK in 2017,^1^ of which most patients were diagnosed with late-stage disease (III or IV).^2^ Achieving earlier diagnosis is critical to reducing lung cancer mortality, because survival from early stage disease is markedly higher (82% 5-year survival for stage IA non-small cell).^3^ The US National Lung Screening Trial and the Dutch-Belgian Nederlands–Leuvens Longkanker Screenings Onderzoek (NELSON) trial have shown that screening high-risk, asymptomatic adults for early stage lung cancer using low-dose CT (LDCT) reduces the relative risk of lung cancer mortality by 20% and 24%, respectively.^4 5^ Consequently, LDCT screening is recommended in the USA, some regions of China, Korea and Croatia and the UK’s National Screening Committee are reviewing the recently published NELSON trial results.

Central to policy decision-making about population screening programmes is ensuring the benefit of screening for the few (ie, averted cancer deaths) outweighs any potential harm caused to the whole screened population.^6^ This includes psychological harm, which may be especially likely among those receiving abnormal results. Some earlier LDCT screening trials found a relatively high rate of false positive and incidental results, with one review estimating an average pulmonary nodule detection rate of 20% (range 3%–51%).^7^ However, changes to the way nodules are categorised mean the NELSON trial’s false-positive rate was substantially reduced to 1.2%.^5^

Nevertheless, research has sought to determine whether LDCT screening and the different types of screening result cause psychological morbidity. In the short term, participants with abnormal findings reported lower health-related quality of life (HRQoL) in the NELSON trial^8^ and increased psychological distress in the UK Lung Screening (UKLS) trial,^9^ when compared with participants receiving negative results. However, with the exception of individuals who received a lung cancer diagnosis, no clinically significant consequences for psychological well-being or HRQoL were observed in the long-term across USA and European screening trials when compared with the control trial arms.^10 11^ While reassuring, evidence suggests a minority experience clinically significant increases in anxiety^12^ and that particular characteristics could confer greater propensity for distress. In the UKLS trial, female gender, younger age (<65 years), study site (relatively deprived vs affluent) and current smoking status were associated with increased distress in both the screening and control arms.^9^ This potential association of current smoking status and deprivation with increased distress is important because these same characteristics predict lower uptake of LDCT screening trials,^13–15^ meaning these characteristics are relatively underrepresented in studies to date. Furthermore, the finding that distress was elevated among these subgroups even within the ‘unscreened’ control arm is similar to that of the Danish Lung Screening Trial, which observed negative psychological outcomes in both trial arms.^16^ Control arm trial participants are told they are at high enough risk to enrol, yet not offered screening. They may therefore be more distressed than those who are screening naïve, making them an inappropriate comparison group and potentially underestimating screening-induced distress.

The external validity of psychological outcome data from LDCT screening trials may therefore be limited due both to low participation by those subgroups reporting higher distress and to elevated distress within the ‘no screen’ control arm with which screening participants’ psychological outcomes are often compared. Ours is the first study to compare psychological outcomes among individuals who had undergone LDCT screening in a real-world demonstration pilot, with a community comparison sample who had never been offered LDCT screening. The specific aims were to (1) investigate the sociodemographic and smoking-related characteristics associated with psychological outcomes following screening and (2) compare the immediate and short-term psychological outcomes of screened individuals with those of the screening unaware community comparison sample both overall and by LDCT screening result.

## Methods

### Screening cohort sample

Recruitment was nested within the Lung Screen Uptake Trial (LSUT^17 18^); a real-world demonstration pilot of LDCT screening across two diverse London sites, which aimed to improve uptake and reduce socioeconomic and smoking-related inequalities in participation. Potentially eligible individuals were invited to attend a prescheduled Lung Health Check (LHC) appointment via postal invitation letters from their general practitioner (see Quaife *et al* 17 for detailed invitation methods). One thousand and five current and former smokers (quit <7 years), aged 60–75, underwent a LHC hospital appointment at which LDCT screening was offered to those eligible (n=845) on the same day. Regardless of LDCT eligibility, all participants were asked to self-complete paper questionnaires containing validated psychological instruments at three time points: their LHC appointment (T_0_), the next day (T_1_) and at 3 month follow-up (T_2_). The latter time point was chosen both because all participants would have received their LDCT results and because any participant requiring a follow-up appointment would have had this within 3 months of their appointment. Part way through the study, reminder letters and a prize draw were introduced to improve response rate at T_2_.

### Community comparison sample

Four hundred participants who had not been invited to screening, but shared the same age (60–75 years) and smoking characteristics (current or former smoker quit <7 years) as the screening sample, were recruited via the Smoking Toolkit Study (STS).^19^ The STS collects monthly national data on smoking behaviour of current and former smokers in England within Ipsos MORI’s (market & opinion research international) face-to-face Omnibus survey. Ipsos MORI uses a nationally representative random location sampling design and home-based computer-assisted interviewing. Participants self-completed the psychological outcome measures at one time point (T_0_) using an electronic tablet. LDCT screening was not mentioned.

## Measures

### Psychological outcomes

Anxiety and depression were measured using the Hospital Anxiety and Depression Scale (HADS).^20^ Participants were asked to indicate how they felt during the last week on a 14-item scale with four response options (scored 0–3). Scores for the anxiety and depression scales were summed separately (range 0–21) and interpreted using clinical thresholds: normal (range 0–7), mild (range 8-10) moderate (range 11-14) and severe (range 15-21).^20^

Lung cancer worry was measured using an adapted version of the Cancer Worry Scale.^21^ This included seven items with 4-point or 5-point response scales. Total scores were summed (range: 7–29), with higher scores representing higher worry.

Aggregate mean scores for cancer worry, anxiety and depression were then computed at each respective time point.

### Sociodemographic and smoking-related characteristics

For the screening sample, current smoking status, age, gender, ethnicity, marital status, employment status and highest level of education were collected during the LHC appointment. For the community sample, these data were obtained via the STS.

### LDCT screening results

LDCT results for the screening sample (from clinical records) were categorised as ‘negative’ (no signs of lung cancer/abnormalities), ‘indeterminate pulmonary nodule’ (requiring 3-month follow-up scan), ‘suspicious thoracic lesion’ (requiring 2-week wait referral), ‘incidental finding requiring general practitioner (GP) follow-up’ or ‘incidental finding requiring hospital follow-up’. There was also a ‘no LDCT scan’ group who were not eligible for LDCT screening.

### Statistical analysis

Analyses of psychological outcomes within the screening sample were prespecified within a prospectively registered statistical analysis plan (https://osf.io/hkemm). This was followed except for analysis by LSUT arm, because there was no overall effect of the intervention on uptake. Further funding was awarded to collect additional data from a community comparison sample. Analyses were prespecified within the funding application, but were not openly registered.

Descriptive analyses compared the sociodemographic characteristics and smoking status of the two samples and those within the screening sample who completed the questionnaire measures and those who did not. The latter comparison also included LDCT result. Independent sample t tests and χ^2^ tests explored potential differences.

Analyses tested for differences in mean scores for cancer worry, anxiety and depression by sociodemographic characteristics and smoking status, using analysis of variance (ANOVA) and independent sample t tests. The screening sample’s overall mean scores on each psychological outcome at each time point (T_0_, T_1_ and T_2_) were then compared with those of the community sample (T_0_) using ANOVA. These analyses were repeated to explore differences in mean scores by LDCT result specifically, with Tukey post hoc comparisons. Multivariable linear regression analyses then tested whether sample type and LDCT result predicted each of the psychological outcomes independent of sociodemographic characteristics and smoking status.

Additional analyses (not in the prespecified plan and reported in online supplemental tables) determined the proportion of participants who scored above the clinical thresholds (>11) for moderate/severe anxiety and depression (vs below this threshold, that is, mild/normal) on the HADS measure. We examined these proportions within each sociodemographic and LDCT screening result subgroup and conducted multivariable logistic regression models to test the independence of these associations when adjusted for sociodemographic characteristics and smoking status.

10.1136/thoraxjnl-2020-215054.supp1 Supplementary data

All analyses were performed in SPSS (V.25) using a complete-case approach. All multivariable analyses of T_1_ and T_2_ outcomes were adjusted for T_0_ scores. Due to multiple testing, a more stringent alpha level of 0.01 was used. Sensitivity analyses excluded participants who had completed the questionnaire outside the expected timeframes (T_0_=same day, T_1_=<2 weeks, T_2_=3–5 months). Cognisant of the fact that psychological scores can have skewed distributions, distributions were checked, and positive skewness was found in the cancer worry, anxiety and depression scores at T_0_. Multivariable regression analyses were carried out on the log-transformed scores, which found qualitatively the same results. The results are presented in the original scale, as the differences these describe are more readily interpretable.

### Statistical power

We anticipated a priori that 700 screening participants would complete the baseline measure and 45% (n=315) would return the follow-up measures based on previous research.^22^ A quota of 400 participants was set for the community comparison sample, in that, 315 screening participants and 400 community controls provide >80% statistical power to detect small between-group and within-group differences (d=0.2) using two-tailed tests and including eight predictors in multivariable regression modelling (f^2^=0.05).

## Results

### Sample characteristics

At T_0_, both samples had a similar proportion of men (54%) and average age of 66 years (see table 1). Relative to the community sample, the screening sample was more ethnically diverse, more frequently retired, more commonly married/cohabiting and reported lower education (all p’s<0.01). A smaller proportion of the screening sample was current smokers (69% vs 81% in community sample, p<0.001).

**Table 1:** Comparison of sociodemographic characteristics between those completing each psychological outcome measure in the screening sample (T_0_) and the community sample

	Cancer worry			Anxiety			Depression		
	Community sample (n=383)	Screening sample (n=787)	P value	Community sample (n=376)	Screening sample (n=744)	P value	Comparison sample (n=384)	Screening sample (n=755)	P value
Gender, n (%)									
Female	176 (46.0)	362 (46.0)	0.99*	172 (44.8)	351 (47.2)	0.45*	174 (45.3)	349 (46.2)	0.77*
Male	207 (54.0)	425 (54.0)		212 (55.2)	393 (52.8)		210 (54.7)	406 (53.8)	
Age, mean (SD*)*	66.24 (*4.18*)	65.75 (*4.01*)	0.06†	66.69 (*4.42*)	65.75 (*4.05*)	0.05†	66.47 (*4.46*)	65.83 (*4.06*)	<0.05†
Ethnicity, n (%)									
White	367 (96.3)	660 (84.1)	<0.001*	366 (95.8)	632 (85.2)	<0.001*	367 (96.1)	640 (84.9)	<0.001*
Minority ethnic group	14 (10.1)	125 (15.9)		16 (4.2)	110 (14.8)		15 (3.9)	114 (15.1)	
Education, n (%)									
Finished school ≤age of 15	119 (31.3)	387 (49.2)	<0.001*	121 (31.5)	363 (48.9)	<0.01*	120 (31.3)	361 (47.9)	<0.001*
Completed CSEs/O levels	108 (28.2)	83 (10.5)		107 (27.9)	78 (10.5)		106 (27.6)	78 (10.3)	
Completed A levels/further/other	95 (24.8)	138 (17.5)		97 (25.3)	130 (17.5)		96 (25.0)	138 (18.3)	
Completed university degree	61 (15.9)	179 (22.7)		59 (15.4)	172 (23.1)		62 (16.1)	177 (23.5)	
Employment status, n (%)									
Retired	265 (69.2)	481 (62.7)	<0.01*	268 (69.8)	453 (62.7)	<0.001*	268 (69.8)	460 (62.8)	<0.01*
Employed	78 (20.4)	226 (29.5)		75 (19.5)	217 (30.0)		76 (19.8)	220 (30.0)	
Unemployed/disabled/homemaker/other	40 (10.4)	60 (7.8)		41 (10.7)	53 (7.3)		40 (10.4)	53 (7.2)	
Marital status, n (%)									
Married/cohabiting	203 (53.1)	355 (45.2)	<0.01*	208 (54.3)	329 (44.3)	<0.01*	205 (53.5)	338 (44.9)	<0.01*
Not married/cohabiting	179 (46.9)	431 (54.8)		175 (45.7)	413 (55.7)		178 (46.5)	415 (55.1)	
Smoking status, n (%)									
Current smoker (including occasional)	308 (80.8)	538 (68.6)	<0.001*	308 (80.6)	511 (68.9)	<0.001*	308 (80.6)	515 (68.5)	<0.001*
Former smoker	72 (19.2)	246 (31.4)		74 (19.4)	231 (31.1)		74 (19.4)	237 (31.5)	

Ns may vary in each cell due to missing data.

*χ^2^ test (categorical variables).

†Independent samples t-test (continuous variables).

CSE, Certificate of Secondary Education.

### Response rates

Response rates were unknown for the community comparison sample but missing data among respondents were low (1.0%, n=17).

For the screening sample, 82.5% (n=829) completed the questionnaire at T_0_, 51.6% at T_1_ (n=519) and 43.1% at T_2_ (n=433) out of the 1005 LSUT participants attending the LHC. Of those completing the questionnaires, an average of 94.2% had complete data across time points. Table 2 shows the baseline (T_0_) characteristics of ‘completers’ (completing every item) and ‘non-completers’ (including both non-responders and responders who had incomplete/missing data on >1 item) for each psychological outcome measure. Compared with completers, a higher proportion of non-completers had a lower level of education, were unmarried/not cohabiting, were of a black, asian or minority ethnic background and were current (rather than former) smokers (all p’s<0.01, except for response by ethnicity for cancer worry). Non-completers of the cancer worry and anxiety measures were also older on average than completers (~1 year), more frequently ineligible for LDCT screening and less frequently received a negative or indeterminate result (p<0.001). Similar differences were observed at T_1_ and T_2_ (data not reported).

**Table 2:** Comparison of T_0_ completers and non-completers of each psychological outcome in the screening sample

	Cancer worry			Anxiety			Depression		
	T_0_ completers (n=787)	T_0_ non-completers (n=218)	P value	T_0_ completers (n=744)	T_0_ non- completers (n=261)	P value	T_0_ completers (n=755)	T_0_ non- completers (n=250)	P value
Gender, n (%)									
Female	362 (46.0)	94 (43.1)	0.45†	351 (47.2)	105 (40.2)	0.05†	349 (46.2)	107 (42.8)	0.35†
Male	425 (54.0)	124 (56.9)		393 (52.8)	156 (59.8)		406 (53.8)	143 (57.2)	
Age, mean (SD*)*	65.67 (*4.01*)	66.83 (*4.60*)	<0.01‡	65.75 (*4.05*)	66.69 (*4.42*)	<0.01‡	65.83 (*4.06*)	66.47 (*4.46*)	0.05‡
Ethnicity, n (%)									
White	660 (84.1)	168 (77.4)	0.02†	632 (85.2)	196 (75.4)	<0.001†	640 (84.9)	188 (75.8)	<0.01†
Minority ethnic group	125 (15.9)	49 (22.6)		110 (14.8)	64 (24.6)		114 (15.1)	60 (24.2)	
Education status, n (%)									
Finished school ≤age of 15	387 (49.2)	136 (63.0)	<0.01†	363 (48.9)	160 (61.5)	<0.01†	361 (47.9)	162 (65.1)	<0.001†
Completed CSEs/O levels	83 (10.5)	21 (9.7)		78 (10.5)	26 (10.0)		78 (10.3)	26 (10.4)	
Completed A-levels/further/other	138 (17.5)	27 (12.5)		130 (17.5)	35 (13.5)		138 (18.3)	27 (10.8)	
Completed university degree	179 (22.7)	32 (14.8)		172 (23.1)	39 (15.0)		177 (23.5)	34 (13.7)	
Employment status, n (%)									
Retired	481 (62.7)	140 (68.0)	0.36†	453 (62.7)	168 (67.2)	0.18†	460 (62.8)	161 (67.1)	0.14†
Employed	226 (29.5)	51 (24.8)		217 (30.0)	60 (24.0)		220 (30.0)	57 (23.8)	
Unemployed/disabled/homemaker/other	60 (7.8%)	15 (7.3%)		53 (7.3)	22 (8.8)		53 (7.2)	22 (9.2)	
Marital status, n (%)									
Married/cohabiting Not married/cohabiting	355 (45.2) 431 (54.8)	73 (33.8) 143 (66.2)	<0.01†	329 (44.3) 413 (55.7)	99 (38.1) 161 (61.9)	0.08†	338 (44.9) 415 (55.1)	90 (36.1) 159 (63.9)	0.02†
Smoking status, n (%)									
Current smoker (including occasional)	538 (68.6)	171 (79.2)	<0.01†	511 (68.9)	198 (76.7)	0.02†	515 (68.5)	194 (78.2)	<0.01†
Former smoker	246 (31.4)	45 (20.8)		231 (31.1)	60 (23.3)		237 (31.5)	54 (21.8)	
LDCT scan result, n (%)									
No LDCT scan	156 (19.8)	79 (36.2)	<0.001†	142 (19.1)	93 (35.6)	<0.001†	150 (19.9)	85 (34.0)	<0.001†
Negative LDCT scan	196 (24.9)	41 (18.8)		187 (25.1)	50 (19.2)		189 (25.0)	48 (19.2)	
Indeterminate pulmonary nodule	104 (13.2)	23 (10.6)		107 (14.4)	20 (7.7)		105 (13.9)	22 (8.8)	
Suspicious thoracic lesion	27 (3.4)	6 (2.8)		23 (3.1)	10 (3.8)		27 (3.6)	6 (2.4)	
Incidental finding (GP follow-up)	268 (34.1)	60 (27.5)		251 (33.7)	77 (29.5)		250 (33.1)	78 (31.2)	
Incidental finding (hospital follow-up)	36 (4.6)	9 (4.1)		34 (4.6)	11 (4.2)			11 (4.4)	

Ns may vary in each cell due to missing data.

*χ^2^ test (categorical variables)

†Independent samples t test (continuous variables)

CSE, Certificate of Secondary Education; GP, General Practitioner; LDCT, low-dose CT.

The majority of respondents completed their T_0_ survey on the same day as their appointment (85.8%), their T_1_ survey within 2 weeks of their appointment (90.5%) and their T_2_ survey within 3–5 months of their appointment (91.8%).

### Sociodemographic and smoking-related differences in psychological outcomes within the screening sample

There were few statistically significant differences in baseline psychological outcomes by sociodemographic characteristics, none by smoking status, and none at T_1_ or T_2_ after adjusting for sociodemographic factors and baseline psychological outcome score (table 3).

**Table 3:** Mean psychological outcome scores for the screening sample by sociodemographic characteristics and smoking status

	Cancer worry T_0_ (range 7–29)	Cancer worry T_1_ (range 7–29)	Cancer worry T_2_ (range 7–29)	Anxiety T_0_ (range 0–21)	Anxiety T_2_ (range 0–21)	Depression T_0_ (range 0–21)	Depression T_2_ (range 0–21)
	Mean (95% CI)	Mean (95% CI)	Mean (95% CI)	Mean (95% CI)	Mean (95% CI)	Mean (95% CI)	Mean (95% CI)
Gender†							
Female	11.79 (11.40 to 12.18)**	11.31 (10.84 to 11.78)	12.05 (11.46 to 12.64)	5.61 (5.12 to 6.10)**	6.40 (5.71 to 7.10)*	3.61 (3.21 to 4.00)	4.16 (3.57 to 4.75)
Male	10.95 (10.63 to 11.27)**	10.66 (10.25 to 11.07)	11.73 (11.21 to 12.26)	3.95 (3.56 to 4.33)**	5.25 (4.66 to 5.83)*	3.06 (2.73 to 3.39)	4.15 (3.60 to 4.69)
Age, beta (95% CI)‡	−0.04 (-0.10 to 0.03)	−0.08 (-0.15 to -0.00)	−0.06 (-0.15 to 0.04)	−0.11 (-0.18 to -0.03)**	−0.22 (-0.32 to -0.11)*	−0.08 (-0.13 to -0.01)	−0.08 (-0.18 to 0.02)
Ethnicity§							
White	11.38 (11.11 to 11.64)	11.09 (10.76 to 11.42)	11.77 (11.37 to 12.17)	4.79 (4.45 to 5.14)	5.79 (5.32 to 6.25)	3.21 (2.94 to 3.50)	4.08 (3.67 to 4.49)
Minority ethnic group	11.15 (10.42 to 11.88)	10.09 (9.15 to 11.02)	13.03 (11.31 to 14.74)	4.44 (3.69 to 5.19)	5.67 (3.88 to 7.46)	3.88 (3.26 to 4.49)	5.00 (3.39 to 6.61)
Education§							
Left school ≤age 15	11.45 (11.06 to 11.85)	10.82 (10.35 to 11.30)	12.23 (11.60 to 12.86)	4.85 (4.39 to 5.31)	6.33 (5.61 to 7.04)	3.60 (3.25 to 3.95)*	5.02 (4.41 to 5.64)*
CSEs/O levels	10.95 (10.22 to 11.68)	10.77 (9.93 to 11.61)	12.02 (10.76 to 13.28)	4.40 (3.45 to 5.34)	5.89 (4.35 to 7.44)	3.85 (2.93 to 4.76)*	4.26 (2.77 to 5.75)*
A levels/further/other	11.54 (10.98 to 12.10)	11.43 (10.58 to 12.28)	12.14 (11.21 to 13.08)	5.10 (4.33 to 5.87)	6.00 (5.02 to 6.98)	3.10 (2.49 to 3.72)*	3.70 (2.94 to 4.45)*
University degree	11.10 (10.63 to 11.57)	11.04 (10.48 to 11.60)	11.05 (10.41 to 11.70)	4.34 (3.72 to 4.96)	4.73 (3.96 to 5.50)	2.64 (2.12 to 3.17)*	3.04 (2.32 to 3.75)*
Employment status§							
Retired	11.46 (11.12 to 11.81)	11.11 (10.67 to 11.55)	12.27 (11.76 to 12.78)	4.80 (4.39 to 5.22)*	5.98 (5.40 to 6.55)**	3.52 (3.19 to 3.86)**	4.62 (4.10 to 5.14)*
Employed	11.19 (10.79 to 11.60)	10.73 (10.31 to 11.15)	11.10 (10.42 to 11.77)	4.03 (3.52 to 4.53)**	5.07 (4.31 to 5.84)**	2.40 (2.02 to 2.79)**	2.73 (2.14 to 3.31)*
Unemployed/disabled/homemaker/other	11.07 (10.05 to 12.08)	11.23 (9.87 to 12.59)	12.00 (10.27 to 13.73)	6.87 (5.60 to 8.14)**	7.92 (5.97 to 9.87)**	5.72 (4.94 to 6.94)**	5.96 (4.15 to 7.78)*
Marital status§							
Married/cohabiting	11.17 (10.82 to 11.52)	10.95 (10.49 to 11.42)	11.64 (11.12 to 12.17)	4.38 (3.91 to 4.85)	5.35 (4.72 to 5.98)	2.86 (2.49 to 3.23)**	3.33 (2.81 to 3.84)*
Not married/cohabiting	11.48 (11.13 to 11.84)	10.99 (10.57 to 11.41)	12.07 (11.50 to 12.64)	5.01 (4.59 to 5.43)	6.14 (5.51 to 6.77)	3.68 (3.33 to 4.03)*	4.82 (4.25 to 5.40)*
Smoking status†							
Current smoker¶	11.37 (11.07 to 11.67)	11.10 (10.72 to 11.48)	12.07 (11.58 to 12.55)	4.76 (4.38 to 5.14)	6.02 (5.44 to 6.59)	3.39 (3.08 to 3.70)	4.50 (3.98 to 5.02)
Former smoker	11.29 (10.82 to 11.76)	10.75 (10.19 to 11.30)	11.53 (10.86 to 12.20)	4.64 (4.07 to 5.20)	5.37 (4.65 to 6.09)	3.16 (2.70 to 3.60)	3.55 (2.95 to 4.15)

*p<0.01 in unadjusted analyses, **p<0.01 (bold) in unadjusted analyses and linear regression models adjusted for gender, age, ethnicity, education, employment status, marital status and smoking status. For psychological outcomes at T_1_ and T_2_, the models were also adjusted for T_0_ outcomes.

*Independent samples t-test.

†Bivariate linear regression.

‡One-way ANOVA.

§Including occasional smokers.

CSE, Certificate of Secondary Education.

For cancer worry, women had a higher mean score (mean (M): 11.79; 95% CI 11.40 to 12.18) than men (M: 10.95; 10.63 to 11.27 p<0.01) at T_0_ in unadjusted and adjusted analyses, but the absolute difference was small.

For anxiety, women again reported higher mean levels compared with men at both T_0_ (M: 5.61; 5.12 to 6.10 vs M: 3.95; 3.56 to 4.33, respectively, p<0.001) and T_2_ (M: 6.40; 5.71 to 7.10 vs M: 5.25; 4.66 to 5.83, p<0.01) in unadjusted analyses. Women were also more likely to score above the threshold for moderate/severe anxiety at T_0_ than men (adjusted OR (aOR): 2.83; 1.70 to 4.71, p<0.001, see online supplemental table 1). The mean scores for both men and women remained within the ‘normal’ clinical range and differences were no longer statistically significant at T_2_ in adjusted analyses of both mean scores and dichotomised scores (normal/mild vs moderate/severe). Younger age was also associated with higher anxiety at both these time points (T_0_ B:−0.11;−0.18 to −0.03, T_2_ B:−0.22;−0.32 to −0.11, p’s<0.01) in unadjusted analyses, as was employment status. For example, participants, who were unemployed/disabled/homemakers, had significantly higher mean anxiety scores at T_2_ (M: 7.92; 5.97 to 9.87) than those who were employed (M: 5.07; 4.31 to 5.84) or retired (M: 5.98; 5.40 to 6.55, p<0.001). In this instance, these differences were clinically meaningful, because those in the unemployed/disabled/homemaker group had a mean anxiety score within the ‘mild’ clinical range. However, in the adjusted analyses, the differences by age and employment were no longer statistically significant at T_2_ and in multivariable logistic regression analyses, these groups were no more likely to score above the cut-off for moderate/severe anxiety at either T_0_ or T_2_ (online supplemental table 1).

For depression, the pattern by employment status was similar to that of anxiety. At T_2_, those who reported being unemployed/disabled/homemakers had a statistically significantly higher mean depression score (M: 5.96; 4.15 to 7.78) in unadjusted analyses compared with those who were employed (M: 2.73; 2.14 to 3.31) or retired (M: 4.62; 4.10 to 5.14, p<0.01). Further analyses (online supplemental table 2) also showed that an ‘unemployed/disabled/homemaker’ status (vs retired) increased the odds of scoring above the threshold for moderate/severe depression at T_0_ (aOR: 3.19; 1.39 to 7.35, p<0.01) while older age reduced the odds (aOR: 0.86; 0.78 to 0.96, p<0.01). Having less education was also associated with higher depression scores at both time points in unadjusted analyses (eg, left school <15 T_2_ M: 5.02; 4.41 to 5.64 vs university degree T_2_ M: 3.04; 2.32 to 3.75, p<0.01). In addition, those who were married/cohabiting reported lower depression scores at T_0_ (M: 2.86; 2.49 to 3.23) and T_2_ (M: 3.33; 2.81 to 3.84) than those who were not married/cohabiting (M: 3.68; 3.33 to 4.03 and M: 4.82; 4.25 to 5.40 at T_0_ and T_2_, respectively). Despite these differences, all mean scores for depression remained within the ‘normal’ clinical range. Furthermore, in adjusted analyses, these differences and associations were no longer statistically significant at T_2_.

### Overall differences in psychological outcomes between the screening and community samples

In unadjusted analyses, the screening sample had statistically significantly higher mean cancer worry scores at all time points (T_0_ M: 11.34; 11.09 to 11.59; T_1_ M: 10.97; 10.66 to 11.28; T_2_ M: 11.88; 11.49 to 12.27) than the community sample at T_0_ (M: 9.32; 8.96 to 9.69, all p’s<0.001), although absolute differences were small (~2; table 4). In analyses adjusted for sociodemographic characteristics, smoking status and baseline (T_0_) cancer worry score, this association was no longer significantly higher at T_1_.

**Table 4:** Multivariable linear regression predicting psychological outcomes for the screening sample compared with the community sample

	Community sample	Screening sample	P value	Estimate (adjusted)*	
	Estimate (unadjusted)				
	Mean (95% CI)	Mean (95% CI)		Beta (95% CI)	P value
Cancer worry T_0_	9.32 (8.96 to 9.69)	11.34 (11.09 to 11.59)	<0.001	1.99 (1.51 to 2.64)	<0.001
Cancer worry T_1_		10.97 (10.66 to 11.28)	<0.001	0.08 (-0.19 to 0.34)	0.56
Cancer worry T_2_		11.88 (11.49 to 12.27)	<0.001	0.87 (0.49 to 1.25)	<0.001
Anxiety T_0_	3.32 (2.94 to 3.70)	4.73 (4.42 to 5.04)	<0.001	1.38 (0.85 to 1.92)	<0.001
Anxiety T_2_		5.78 (5.33 to 6.23)	<0.001	1.33 (0.99 to 1.68)	<0.001
Depression T_0_	3.85 (3.44 to 4.27)	3.32 (3.06 to 3.57)	0.02	−0.51 (-0.99 to -0.03)	0.04
Depression T_2_		4.15 (3.76 to 4.55)	0.30	0.64 (-0.32 to 0.95)	<0.001

Score ranges for each psychological outcome measure are: cancer worry (7-29), anxiety (0–21), depression (0–21); models adjusted for gender, age, ethnicity, education, employment status, marital status and smoking status. For psychological outcomes at T_1_ and T_2_, the models were also adjusted for T_0_ outcomes.

The screening sample also had higher mean anxiety scores at T_0_ (M: 4.73; 4.42 to 5.04) and T_2_ (M: 5.78; 5.33 to 6.23) than the community sample at T_0_ (M: 3.32; 2.94 to 3.70), in unadjusted and adjusted analyses (all p’s<0.001). Again, absolute differences were small (~2) and scores remained with the ‘normal’ clinical range for anxiety. For depression, a statistically significant difference between samples was only observed in adjusted analyses at T_2_ (M: 4.15; 3.76 to 4.55 vs M: 3.85; 3.44 to 4.27, p<0.001) and not T_0_. The absolute difference was 0.3 and all scores were within the ‘normal’ clinical range.

### Differences in psychological outcomes between the screening and community samples by LDCT result

Mean scores for cancer worry at T_2_ among the screening sample were significantly higher for all but one (incidental findings requiring hospital follow-up) of the LDCT result subgroups at T_0_ when compared with the community sample at T_0_ (table 5). Except for those receiving a negative LDCT result, cancer worry scores were highest at T_2_ and significantly higher across all the screening subgroups compared with the community sample at T_0_, including those who had not been screened (M: 12.03; 10.70 to 13.36 vs M: 9.32; 8.96 to 9.69, p<0.001). In analyses adjusted for sociodemographic characteristics, smoking status and T_0_ worry score, receiving an indeterminate result (B: 2.06; 1.37 to 2.76), an incidental finding (GP (B: 0.82; 0.32 to 1.33) and hospital (B: 2.41; 1.15 to 3.66) follow-up) or not being screened (B: 1.31; 0.62 to 2.00) were associated with statistically significantly higher cancer worry scores at T_2_ relative to the community sample at T_0_ (p<0.01; table 6).

**Table 5:** Differences in mean scores on psychological outcomes between the screening and community comparison sample by type of LDCT result

	Community sample (ref)	LSUT sample by screening result						
		Negative LDCT scan	Indeterminate pulmonary nodule	Suspicious thoracic lesion	Incidental finding GP follow-up	Incidental finding hospital follow-up	No LDCT scan	P value
Cancer worry T_0_, mean (95% CI)	9.32 (8.96 to 9.69)	11.81** (11.27 to 12.35)	11.04** (10.36 to 11.71)	12.11* (10.71 to 13.51)	11.25** (10.86 to 11.65)	10.86 (9.80 to 11.93)	11.06** (10.45 to 11.68)	<0.001
Cancer worry T_1_, mean (95% CI)		11.37** (10.77 to 11.98)	11.00* (10.28 to 11.72)	11.42 (9.58 to 13.26)	10.84** (10.31 to 11.38)	11.20 (9.35 to 13.05)	10.14 (9.32 to 10.96)	<0.001
Cancer worry T_2_, mean (95% CI)		11.24** (10.59 to 11.89)	12.97** (11.96 to 13.98)	12.95* (11.03 to 14.87)	11.52** (10.92 to 12.12)	13.05* (10.80 to 15.30)	12.03** (10.70 to 13.36)	<0.001
Anxiety T_0_, mean (95% CI)	3.32 (2.94 to 3.70)	5.25** (4.54 to 5.96)	4.37 (3.56 to 5.19)	5.17 (3.79 to 6.56)	4.49* (3.97 to 5.01)	3.47 (2.31 to 4.63)	4.96* (4.24 to 5.68)	<0.001
Anxiety T_2_, mean (95% CI)		5.29** (4.38 to 6.21)	6.93** (5.65 to 8.21)	6.39 (4.04 to 8.74)	5.34** (4.67 to 6.02)	4.94 (2.93 to 6.95)	6.51** (5.25 to 7.77)	<0.001
Depression T_0_, mean (95% CI)	3.85 (3.44 to 4.27)	3.58 (3.04 to 4.12)	3.21 (2.51 to 3.91)	3.89 (2.42 to 5.36)	3.28 (2.85 to 3.72)	2.65 (1.63 to 3.66)	3.15 (2.58 to 3.73)	0.23
Depression T_2_, mean (95% CI)		3.25 (2.49 to 4.02)	4.81 (3.67 to 5.96)	5.28 (2.71 to 7.84)	3.93 (3.35 to 4.51)	3.33 (1.73 to 4.93)	5.38 (4.25 to 6.51)	0.01

Score ranges for each psychological outcome measure are: cancer worry (7–29), anxiety (0–21), depression (0–21).

*p<0.01 for Tukey HSD posthoc test, **p<0.001 for Tukey HSD posthoc test.

GP, General Practitioner; HSD, honest significant difference; LDCT, low-dose CT.

**Table 6:** Multivariable linear regression predicting psychological outcomes following LDCT screening for the screening sample compared with the community sample

	Community sample	Negative LDCT scan	Indeterminate nodule	Suspicious thoracic lesion	Incidental finding (GP)	Incidental finding (hospital)	No LDCT scan
	Beta (95% CI)	Beta (95% CI)	Beta (95% CI)	Beta (95% CI)	Beta (95% CI)	Beta (95% CI)	Beta (95% CI)
Cancer worry T_2_ (n=748)	Ref	−0.21 (−0.75 to 0.34)	2.06** (1.37 to 2.76)	1.26 (0.06 to 2.46)	0.82* (0.32 to 1.33)	2.41** (1.15 to 3.66)	1.31** (0.62 to 2.00)
Anxiety T_2_ (n=706)	Ref	0.75* (0.23 to 1.26)	1.87** (1.23 to 2.51)	1.15 (−0.06 to 2.37)	1.32** (0.84 to 1.79)	1.36 (0.13 to 2.59)	2.05** (1.35 to 2.75)
Depression T*_2_* (n=706)	Ref	0.09 (−0.39 to 0.56)	1.02* (0.42 to 1.62)	0.60 (−0.44 to 1.64)	0.59* (0.15 to 1.03)	0.04 (−1.05 to 1.14)	1.57** (0.95 to 2.19)

Score ranges for each psychological outcome measure are: cancer worry (7-29), anxiety (0–21), depression (0–21); models adjusted for gender, age, ethnicity, education, employment status, marital status, smoking status and T_0_ psychological outcome scores.

*p<0.01, **p<0.001.

GP, General Practitioner; LDCT, low-dose CT.

For anxiety, participants with a negative LDCT result, an incidental finding requiring GP follow-up or who had not been screened had significantly higher mean scores at T_0_ and T_2_ compared with the community sample in unadjusted and adjusted analyses. Participants found to have an indeterminate pulmonary nodule also had statistically significantly higher anxiety at T_2_ than those in the community sample (M: 6.93; 5.65 to 8.21 vs M: 3.32; 2.94 to 3.70, p<0.001), but not at T_0_. There were no statistically significant differences in anxiety at either T_0_ or T_2_ for those with a suspicious thoracic lesion or an incidental finding needing hospital follow-up. As with cancer worry, mean anxiety scores were highest at T_2_ for all screening result subgroups except those with a negative LDCT result. However, all mean scores remained within the ‘normal’ clinical range.

In unadjusted analyses, there were no statistically significant differences in either T_0_ or T_2_ mean depression scores when comparing each of the screening result subgroups with the community comparison sample at T_0_. However, in adjusted analyses, having an indeterminate pulmonary nodule (B: 1.02; 0.42 to 1.62), an incidental finding requiring GP follow-up (B: 0.59; 0.15 to 1.03) or not being screened (B: 1.57; 0.95 to 2.19) were associated with higher depression scores at T_2_ (all p’s<0.01) relative to the community sample at T_0_. Mean scores for each subgroup remained within clinically normally ranges; however, further analyses showed that those with a suspicious thoracic lesion were significantly more likely to report moderate/severe depression at T_2_ (aOR: 17.61; 2.26 to 137.00, p<0.01, see online supplemental table 3).

## Discussion

This is the first study to investigate psychological outcomes among LDCT screening participants in a real-world demonstration pilot service. We compared scores for anxiety, depression and cancer worry with those of a community sample of ‘screening unaware’ individuals; thus, eliminating any potential psychological impact of screening invitation within the comparison group. There was no evidence that screening participation had a clinically significant impact on psychological well-being. Nevertheless, differences by type of screening result, eligibility status and sociodemographic factors suggest potential risk factors for psychological distress.

While within the normal clinical range, mean psychological outcome scores were highest at 3 months follow-up and for those with indeterminate or incidental results. These higher scores were expected given previous research showing similar short-term distress responses to these types of abnormal result.^8 9^ Without any long-term follow-up, it is unknown whether these responses would have decreased over time, but existing research suggests that any adverse impact is likely to be transient.^9–11^ Previous studies have demonstrated the importance of patient-centred and evidence-based communication in minimising surveillance-related anxiety among individuals diagnosed with incidental pulmonary nodules.^23^ Pre-emptively implementing such strategies could minimise any potential for psychological distress and prepare participants psychologically for abnormal screening results. While mean psychological outcomes were not statistically significantly elevated among those with a suspicious thoracic lesion in adjusted analyses, binary logistic regression analyses showed that this group was more likely to report clinically significant moderate/severe depression at T_2_. The smaller number of cases within the abnormal results subgroups at T_2_, and the binary approach to analysis, reduced statistical power meaning we cannot be confident these groups did not experience significantly elevated psychological distress. Further research using real-world data is needed to understand psychological outcomes among screening participants routed through surveillance and urgent referral pathways.

Interestingly, the psychological outcomes of those who received a negative LDCT result were relatively unchanged at 3 months follow-up, whereas the subgroup within the screening sample who were not screened had increased cancer worry, anxiety and depression relative to the community sample. Previous research has shown negative psychosocial consequences of allocation to ‘no screen’ control arms of LDCT screening trials^16 24^ but unlike these participants, those not screened in the present screening sample were predominantly ineligible for screening due to their lower risk of lung cancer. An individual’s perceived personal risk of lung cancer may differ from their objective clinical risk, and this finding suggests that being ineligible could cause a small degree of psychological distress among those with a smoking history who perceive their risk of lung cancer to be high. This is important considering that an individual’s eligibility status can change over time and suggests that LDCT screening eligibility needs careful communication at both the population and individual level.

Unlike previous research, smoking status did not differentiate psychological responses to LDCT screening, although former smokers in this study had quit more recently (<7 years) than in LDCT screening trials (<15 years). However, some of the same sociodemographic predictors of higher short-term psychological distress^9^ were observed at T_0_. These included female gender and younger age, which were associated not only with increased cancer worry and anxiety but also with lower education, and not being employed or married/cohabiting, which were associated with higher depression (and anxiety in the case of education). However, these differences were not statistically significant at 3 months after adjusting for T_0_ scores. This could suggest that sociodemographic differences are present from the outset when individuals first approach and undergo screening, rather than there being differences in the degree of psychological response to screening. Perhaps the prospect and process of screening evoke more adverse psychological reactions in these groups. Alternatively, this may reflect more widely observed differences in psychological distress and morbidity. Previous research has shown women and lower socioeconomic position (SEP) individuals report higher cancer worry,^25^ that younger age is associated with higher anxiety among patients with cancer,^26^ that education level is inversely associated with anxiety and depression^27^ and that non-married/cohabiting status predicts increased depression.^28^ While no clinically meaningful differences were observed here, further research is needed to establish the origins of poorer psychological outcomes among these subgroups and how these can be improved.

Two important strengths of this study are its external validity and the blinding of the comparison sample to the lung cancer screening context of the study; intended to prevent any potential impact of screening awareness on psychological outcomes. The screening cohort was nested within a screening demonstration pilot across two sites, which aimed to improve uptake and reduce inequalities in participation. This ultimately achieved a sample representative of lower SEP current smokers,^18^ which is important given that these may be risk factors for screening-induced distress.^9 24^ Nevertheless, the present study may still be subject to participation bias. While the aim was to recruit participants with similar demographic and smoking characteristics in both the screening and community comparison samples, their compositions differed on all characteristics except gender and age. These differences were adjusted for statistically and it is reassuring that no clinically meaningful differences were observed despite the comparison sample having characteristics that would be expected to make them more psychologically robust. However, we do not know the relative distribution of lung cancer worry among those in our broader screening-invited population, for those who attended compared with those who did not attend. Worry about risk could have motivated attendance leading to higher reported distress in the screening sample, although evidence-to-date suggests worry about lung cancer risk may actually deter participation so lung cancer worry could be higher among non-attenders.^29^ There were also differences between questionnaire completers and non-completers in the screening sample by ethnicity, education, smoking status and LDCT scan eligibility that may have biased findings. While the absolute amount of missing data was small (~5%), this does further limit the study. Additional limitations are that psychological outcomes were only assessed in the short-term and following a single screen. Participation in a regular screening programme could have a cumulative impact on psychological outcomes that should be studied prospectively and longitudinally in the real-world setting. Finally, response rates to the follow-up surveys (T_1_ and T_2_) were significantly lower than for baseline, which limits the interpretation of the longitudinal analysis.

This study found no clinically significant adverse psychological impact of LDCT screening for lung cancer overall, extending this prior observation from the trial setting to the health service context as well as to a sample predominantly comprised of lower SEP current smokers. In the event of screening implementation, the longitudinal impact of a repeat screening programme across diverse populations and regions within the health service context needs to be researched, as do the differences in psychological response by LDCT result, ineligibility and sociodemographic factors. It is critical that any potential risk factors for distress are better understood and managed pre-emptively through evidence-based, patient-centred communication and screening practice.

## References

[1] Cancer Research UK Lung cancer mortality statistics. Available: https://www.cancerresearchuk.org/health-professional/cancer-statistics/statistics-by-cancer-type/lung-cancer/mortality#heading-Zero

[2] Cancer Research UK Lung cancer incidence statistics. Available: https://www.cancerresearchuk.org/health-professional/cancer-statistics/statistics-by-cancer-type/lung-cancer/incidence#heading-Three

[3] Goldstraw P, Chansky K, Crowley J, et al The IASLC lung cancer staging project: proposals for revision of the TNM stage groupings in the forthcoming (eighth) edition of the TNM classification for lung cancer. J Thorac Oncol 2016;11:39–51. 10.1016/j.jtho.2015.09.009 26762738

[4] National Lung Screening Trial Research Team, Aberle DR, Adams AM, et al Reduced lung-cancer mortality with low-dose computed tomographic screening. N Engl J Med 2011;365:395–409. 10.1056/NEJMoa1102873 21714641 PMC4356534

[5] de Koning HJ, van der Aalst CM, de Jong PA, et al Reduced lung-cancer mortality with volume CT screening in a randomized trial. N Engl J Med 2020;382:503–13. 10.1056/NEJMoa1911793 31995683

[6] Wilson JMG, Jungner G Principles and practice of screening for disease. Available: http://apps.who.int/iris/bitstream/10665/37650/1/WHO_PHP_34.pdf

[7] Bach PB, Mirkin JN, Oliver TK, et al Benefits and harms of CT screening for lung cancer: a systematic review. JAMA 2012;307:2418–29. 10.1001/jama.2012.5521 22610500 PMC3709596

[8] van den Bergh KAM, Essink-Bot ML, Borsboom GJJM, et al Short-Term health-related quality of life consequences in a lung cancer CT screening trial (NELSON). Br J Cancer 2010;102:27–34. 10.1038/sj.bjc.6605459 19935789 PMC2813757

[9] Brain K, Lifford KJ, Carter B, et al Long-Term psychosocial outcomes of low-dose CT screening: results of the UK lung cancer screening randomised controlled trial. Thorax 2016;71:996–1005. 10.1136/thoraxjnl-2016-208283 27471048 PMC5099188

[10] Wu GX, Raz DJ, Brown L, et al Psychological burden associated with lung cancer screening: a systematic review. Clin Lung Cancer 2016;17:315–24. 10.1016/j.cllc.2016.03.007 27130469 PMC5606246

[11] van den Bergh KAM, Essink-Bot ML, Borsboom GJJM, et al Long-Term effects of lung cancer computed tomography screening on health-related quality of life: the NELSON trial. Eur Respir J 2011;38:154–61. 10.1183/09031936.00123410 21148229

[12] Taghizadeh N, Tremblay A, Cressman S, et al Health-Related quality of life and anxiety in the PAN-CAN lung cancer screening cohort. BMJ Open 2019;9:e024719. 10.1136/bmjopen-2018-024719 PMC6340441 30659040

[13] McRonald FE, Yadegarfar G, Baldwin DR, et al The UK Lung Screen (UKLS): demographic profile of first 88,897 approaches provides recommendations for population screening. Cancer Prev Res 2014;7:362–71. 10.1158/1940-6207.CAPR-13-0206 24441672

[14] Aberle DR, Adams AM, et al, National Lung Screening Trial Research Team Baseline characteristics of participants in the randomized National lung screening trial. J Natl Cancer Inst 2010;102:1771–9. 10.1093/jnci/djq434 21119104 PMC2994863

[15] Hestbech MS, Siersma V, Dirksen A, et al Participation bias in a randomised trial of screening for lung cancer. Lung Cancer 2011;73:325–31. 10.1016/j.lungcan.2010.12.018 21324544

[16] Aggestrup LM, Hestbech MS, Siersma V, et al Psychosocial consequences of allocation to lung cancer screening: a randomised controlled trial. BMJ Open 2012;2:e000663. 10.1136/bmjopen-2011-000663 PMC3293139 22382119

[17] Quaife SL, Ruparel M, Beeken RJ, et al The Lung Screen Uptake Trial (LSUT): protocol for a randomised controlled demonstration lung cancer screening pilot testing a targeted invitation strategy for high risk and 'hard-to-reach' patients. BMC Cancer 2016;16:281–282. 10.1186/s12885-016-2316-z 27098676 PMC4839109

[18] Quaife SL, Ruparel M, Dickson JL, et al Lung screen uptake trial (LSUT): randomized controlled clinical trial testing targeted invitation materials. Am J Respir Crit Care Med 2020;201:965–75. 10.1164/rccm.201905-0946OC 31825647 PMC7159423

[19] Fidler JA, Shahab L, West O, et al 'The smoking toolkit study': a national study of smoking and smoking cessation in England. BMC Public Health 2011;11:479. 10.1186/1471-2458-11-479 21682915 PMC3145589

[20] Zigmond AS, Snaith RP The hospital anxiety and depression scale. Acta Psychiatr Scand 1983;67:361–70. 10.1111/j.1600-0447.1983.tb09716.x 6880820

[21] Lerman C, Daly M, Sands C, et al Mammography adherence and psychological distress among women at risk for breast cancer. J Natl Cancer Inst 1993;85:1074–80. 10.1093/jnci/85.13.1074 8515494

[22] Shih T-H, Xitao Fan F. Comparing response rates from web and mail surveys: a meta-analysis. Field methods 2008;20:249–71. 10.1177/1525822X08317085

[23] Slatore CG, Wiener RS Pulmonary nodules: a small problem for many, severe distress for some, and how to communicate about it. Chest 2018;153:1004–15. 10.1016/j.chest.2017.10.013 29066390 PMC5989642

[24] Rasmussen JF, Siersma V, Pedersen JH, et al Psychosocial consequences in the Danish randomised controlled lung cancer screening trial (DLCST). Lung Cancer 2015;87:65–72. 10.1016/j.lungcan.2014.11.003 25433982

[25] Vrinten C, van Jaarsveld CHM, Waller J, et al The structure and demographic correlates of cancer fear. BMC Cancer 2014;14:597. 10.1186/1471-2407-14-597 25129323 PMC4148526

[26] Hinz A, Herzberg PY, Lordick F, et al Age and gender differences in anxiety and depression in cancer patients compared with the general population. Eur J Cancer Care 2019;28:e13129. 10.1111/ecc.13129 31290218

[27] Bjelland I, Krokstad S, Mykletun A, et al Does a higher educational level protect against anxiety and depression? The HUNT study. Soc Sci Med 2008;66:1334–45. 10.1016/j.socscimed.2007.12.019 18234406

[28] Lorant V, Croux C, Weich S, et al Depression and socio-economic risk factors: 7-year longitudinal population study. Br J Psychiatry 2007;190:293–8. 10.1192/bjp.bp.105.020040 17401034

[29] Ali N, Lifford KJ, Carter B, et al Barriers to uptake among high-risk individuals declining participation in lung cancer screening: a mixed methods analysis of the UK lung cancer screening (UKLS) trial. BMJ Open 2015;5:e008254. 10.1136/bmjopen-2015-008254 PMC4513485 26173719

