# Supplementary material for: Psychological outcomes of low-dose CT lung cancer screening in a multisite demonstration screening pilot: the Lung Screen Uptake Trial (LSUT)
Source: Thorax. 2020 Oct 21;75(12):1065–73. doi: 10.1136/thoraxjnl-2020-215054 (PMC7677470; doi:10.1136/thoraxjnl-2020-215054)
Supplement: Supplementary data [file thoraxjnl-2020-215054supp001.pdf]

Supplementary Table 1 Frequencies and multivariable logistic regression for scoring above threshold for moderate/severe anxiety among screening sample

	Anxiety T_0			Anxiety T_2		
	**%(n)**	**%(n)**	**aOR (95% CI)**	**%(n)**	**%(n)**	**aOR (95% CI)**
	**Normal/Mild**	**Moderate/Severe**		**Normal/ Mild**	**Moderate/Severe**	
Gender						
Male	93.6 (368)	6.4 (25)	1.00	88.2 (194)	11.8 (26)	1.00
Female	82.6 (290)	17.4 (61)	**2.83 (1.70,4.71)****	79.8 (150)	20.2 (38)	2.23 (0.97,5.12)
Age	-	-	0.92 (0.86,0.99)	-	-	0.90 (0.79,1.01)
Ethnicity						
White	88.6 (560)	11.4 (72)	1.00	83.7 (313)	16.3 (61)	1.00
Minority ethnic group	87.3 (96)	12.7 (14)	1.23 (0.63,2.41)	90.9 (30)	9.1 (3)	0.20 (0.04,1.09)
Education						
Left school ≤ age 15	88.2 (320)	11.8 (43)	1.00	81.7 (138)	18.3 (31)	1.00
CSEs/O levels	89.7 (70)	10.3 (8)	0.61 (0.25,1.45)	83.0 (39)	17.0 (8)	2.34 (0.68,8.08)
A levels/Further/Other	84.6 (110)	15.4 (20)	1.28 (0.69,2.38)	81.7 (67)	18.3 (15)	0.99 (0.36,2.74)
University degree	91.3 (157)	8.7 (15)	0.81 (0.42,1.56)	90.9 (100)	9.1 (10)	0.32 (0.10,1.06)
Employment status						
Retired	87.6 (397)	12.4 (56)	1.00	82.6 (218)	17.4 (46)	1.00
Employed	93.5 (203)	6.5 (14)	0.42 (0.22,0.81)	89.9 (98)	10.1 (11)	0.58 (0.21,1.57)
Unemployed/Disabled/ Homemaker/Other	75.5 (40)	24.5 (13)	1.53 (0.71,3.27)	76.9 (20)	23.1 (6)	0.31 (0.05,1.82)
Marital status						
Married/cohabiting	89.4 (294)	10.6 (35)	1.00	87.8 (158)	12.2 (22)	1.00
Not married/cohabiting	87.7 (362)	12.3 (51)	1.04 (0.64,1.69)	81.5 (185)	18.5 (42)	1.72 (0.75,3.91)
Smoking status						
Current smoker	88.5 (452)	11.5 (59)	1.00	83.6 (219)	16.4 (43)	1.00
Former smoker	88.7 (205)	11.3 (26)	1.12 (0.67,1.87)	85.5 (124)	14.5 (21)	1.14 (0.50,2.61)

NOTE: aOR = adjusted odds ratio; 95% CI = 95% Confidence Intervals; * p < .01, ** p <.001; Models adjusted for gender, age, ethnicity, education, employment status, marital status, smoking status, and T_0 psychological outcome scores.

Supplementary Table 2 Frequencies and multivariable logistic regression for scoring above threshold for moderate/severe depression among screening sample

	Depression T_0			Depression T_2		
	**%(n)**	**%(n)**	**aOR (95% CI)**			**aOR (95% CI)**
	**Normal/ Mild**	**Moderate/Severe**		**Normal/ Mild**	**Moderate/Severe**	
Gender						
Male	94.6 (384)	5.4 (22)	1.00	93.4 (211)	6.6 (15)	1.00
Female	93.7 (327)	6.3 (22)	0.89 (0.46,1.71)	93.0 (173)	7.0 (12)	0.78 (0.26,2.34)
Age	-	-	**0.86 (0.78,0.96)***	-	-	1.01 (0.86,1.19)
Ethnicity						
White	93.8 (600)	6.3 (40)	1.00	93.4 (351)	6.6 (25)	1.00
Minority ethnic group	96.5 (110)	3.5 (4)	0.47 (0.16,1.42)	91.4 (32)	8.6 (3)	1.11 (0.20,6.07)
Education						
Left school ≤ age 15	95.3 (344)	4.7 (17)	1.00	91.5 (162)	8.5 (15)	1.00
CSEs/O levels	89.7 (70)	10.3 (8)	1.67 (0.63,4.39)	87.0 (40)	13.0 (6)	1.50 (0.31,7.20)
A levels/Further/Other	93.5 (129)	6.5 (9)	1.35 (0.56,3.25)	96.2 (76)	3.8 (3)	0.23 (0.04,1.44)
University degree	94.4 (167)	5.6 (10)	1.54 (0.65,3.61)	96.4 (106)	3.6 (4)	0.25 (0.04,1.47)
Employment status						
Retired	94.6 (435)	5.4 (25)	1.00	91.6 (240)	8.4 (22)	1.00
Employed	97.7 (215)	2.3 (5)	0.26 (0.10,0.72)	98.2 (108)	1.8 (2)	1.00 (0.18,5.60)
Unemployed/Disabled/ Homemaker/Other	75.5 (40)	24.6 (13)	**3.19 (1.39,7.35)***	85.7 (24)	14.3 (4)	1.98 (0.31,12.80)
Marital status						
Married/cohabiting	95.0 (321)	5.0 (17)	1.00	95.6 (175)	4.4 (8)	1.00
Not married/cohabiting	93.5 (388)	6.5 (27)	1.24 (0.64,2.40)	91.2 (208)	8.8 (20)	1.34 (0.42,4.25)
Smoking status						
Current smoker	93.6 (482)	6.4 (33)	1.00	91.6 (240)	8.4 (22)	1.00
Former smoker	95.4 (226)	4.6 (11)	0.76 (0.36,1.61)	96.0 (143)	4.0 (6)	0.75 (0.22,2.55)

NOTE: aOR = adjusted odds ratio; 95% CI = 95% Confidence Intervals; * $p < .01$, ** $p < .001$; Models adjusted for gender, age, ethnicity, education, employment status, marital status, smoking status, and T_0 psychological outcome scores.

Supplementary Table 3 Frequencies and multivariable logistic regression for scoring above threshold for moderate/severe anxiety and depression

	**Community sample**	**Negative LDCT scan**	**Indeterminate Nodule**	**Suspicious Thoracic Lesion**	**Incidental finding (GP)**	**Incidental finding (Hospital)**	**No LDCT scan**
Anxiety T_2							
Normal/Mild, % (n)	94.3 (362)	83.5 (81)	73.3 (44)	77.8 (14)	90.0 (126)	100.0 (16)	81.5 (53)
Moderate/Severe, % (n)	5.7 (22)	16.5 (18)	26.7 (16)	22.2 (4)	10.0 (14)	0.0 (0)	18.5 (12)
Moderate/Severe, aOR	1.00	2.55	3.31	6.80	2.07	-	6.26
(95% CI)		(0.80 to 8.09)	(1.01 to 10.78)	(0.80 to 57.67)	(0.71 to 6.02)	-	(1.54 to 25.43)
Depression T_2							
Normal/Mild, % (n)	91.9 (353)	94.4 (101)	91.5 (54)	77.8 (14)	95.8 (136)	100.0 (18)	89.7 (81)
Moderate/Severe, % (n)	8.1 (31)	5.6 (6)	8.5 (5)	22.2 (4)	4.2 (6)	0.0 (0)	10.3 (7)
Moderate/Severe, aOR	1.00	1.77	0.43	**17.61***	1.08	-	3.06
(95% CI)		(0.38 to 8.19)	(0.05 to 3.46)	**(2.26 to 137.00)**	(0.27 to 4.22)	-	(0.49 to 19.11)

NOTE: aOR = adjusted odds ratio; 95% CI = 95% Confidence Intervals; * p < .01, ** p <.001; Models adjusted for gender, age, ethnicity, education, employment status, marital status, smoking status, and T_0 psychological outcome scores.
